# Shrinkage Properties of Self-Compacting Rubber Lightweight Aggregate Concrete: Experimental and Analytical Studies

**DOI:** 10.3390/ma12244059

**Published:** 2019-12-05

**Authors:** Jing Lv, Tianhua Zhou, Kunlun Li, Kai Sun

**Affiliations:** School of Civil Engineering, Chang’an University, Xi’an 710061, China; zhouth@chd.edu.cn (T.Z.);

**Keywords:** SCRLC, recycling, rubber particles, shrinkage, predictive model

## Abstract

The shrinkage properties of self-compacting rubber lightweight aggregate concrete (SCRLC) were investigated by experimental and analytical studies in this paper. Rubber particles were used to substitute the sand in SCRLC by volume and rubber particles substitution percentages were 10%, 20%, 30%, 40% and 50%. The experimental results showed that the shrinkage strains of SCRLC increased with an increase of rubber particles substitution percentage. On the basis of China Academy of Building Research model and experimental data, a predictive model for describing the shrinkage strains of SCRLC was established. After analytical study, it was revealed that rubber particles substitution percentage of 80% in SCRLC might be the upper limit of substitution percentage; if the rubber particles substitution percentage was larger than 80%, the shrinkage strains of SCRLC would exceed the limiting value stipulated in JGJ51-2002 and the durability of SCRLC would drop to a worse status.

## 1. Introduction

Shrinkage property, one of durability of concrete, significantly affects the application of concrete, especially in mass concrete structures, high performance concrete structures and thin shells. So far, plenty of investigations have been conducted on the different types of shrinkage properties of concrete, such as plastic shrinkage [1], drying shrinkage [2], chemical shrinkage [3], and carbonation shrinkage [4]. Indeed, in most cases, the above different types of shrinkage strains occurred simultaneously and the shrinkage deformation of concrete was the result of a combination of different types of shrinkage strain [5,6]. Thus, in order to achieve a good understanding of the macroscopic shrinkage behavior of concrete, a total shrinkage deformation including drying shrinkage, chemical shrinkage, and carbonation shrinkage was investigated in some literature [7,8,9].

Self-compacting rubber lightweight aggregate concrete (SCRLC) is a new type of lightweight aggregate concrete (LC) with a number of virtues [10,11], typically in recycling waste tires, increasing the toughness of concrete, reducing self-weight of concrete, etc. Nevertheless, previous studies indicate that utilizing rubber particles to substitute sand in normal concrete (NC) will also cause some harmful influences, such as reducing the strength of concrete [12,13], decreasing workability of concrete [14], increasing toughness of concrete [15], increasing shrinkage of concrete [16], etc. It can be deduced that effect of rubber particles on the properties of LC and NC might be similar. Up to now, the effects of rubber particles on the mechanical properties of LC have been verified which are similar to the NC [10,17], whereas few studies are focus on the shrinkage properties of SCRLC. Therefore, it is necessary to conduct the shrinkage test to understand the shrinkage behavior of SCRLC.

As a vital index in consideration of concrete durability, the shrinkage properties of NC have been investigated frequently in recent years [18,19,20]. Based on experimental data, plenty of models are proposed to describe the shrinkage strain of concrete [21,22,23,24,25,26,27,28,29,30]. Although different models consider disparate factors on the effect of shrinkage strains, most of the factors affecting shrinkage strains obviously are considered in existing models. Incorporation of rubber particles and the introduction of self-compacting technology in LC leads to great differences between LC and SCRLC, not only in mechanical properties, but also durability properties. Despite the fact that shrinkage strain of LC can be described by existing models [31,32], using existing models to predict shrinkage strain of SCRLC may be unsuitable. As a new green structural material, it is necessary to have a good understanding on the shrinkage properties of SCRLC for its popularization and application. However, few existing research focuses on the shrinkage properties of SCRLC; the most relevant research report may be the shrinkage properties of rubberized concrete, which is still quite different from SCRLC [13,14,16].

Based on the above background, this paper was undertaken to detect the effect of rubber particles on the shrinkage properties of SCRLC. The rubber particles are utilized to substitute sand by volume, with substitution percentages of 10%, 20%, 30%, 40% and 50%. The contact method is used to evaluate the shrinkage strains of SCRLC. Here, the shrinkage strain is total shrinkage, and the testing ages are 1d, 3d, 7d, 28d, 60d, 90d, 120d, 150d, 180d and 360d. After analyzing the experimental data, combining with contrastive analysis of existing shrinkage predictive models, the shrinkage prediction model of SCRLC is proposed. Then, the shrinkage strains of SCRLC with rubber particles substitution percentages ranged from 60% to 100% are predicted by established model. The limiting rubber particles substitution percentages in SCRLC is also given on account of limiting values of shrinkage strain.

## 2. Experimental Program

### 2.1. Material Properties and Mix Proportions

SCRLC composed of ordinary Portland cement, fly ash, shale ceramsite, sand, rubber particles, thickener, water reducer and water was prepared in this research. In order to investigate the effects of rubber particles on the shrinkage properties of SCRLC, rubber particles were used to substitute sand by volume and set as a unique variable in design of mix proportion. The detail of each mix proportion was exhibited in Table 1. The volume substitution percentages were 10%, 20%, 30%, 40% and 50%.

**Figure 1:**
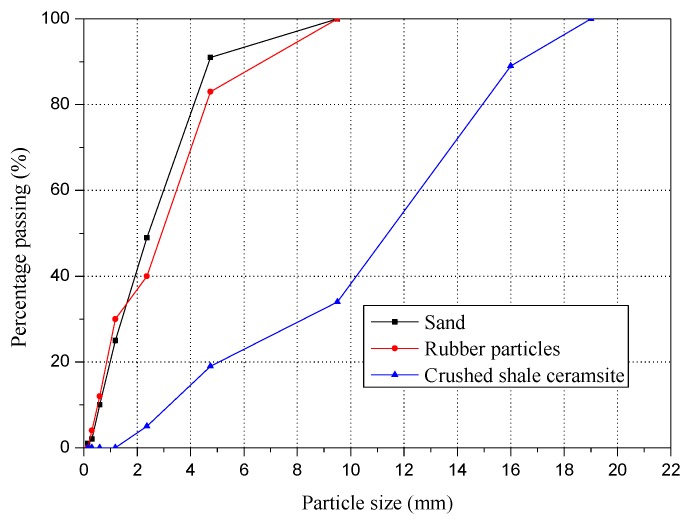
Grading curves of sand, rubber particles and crushed shale ceramsite.

The chemical compositions and physical properties of binding material including ordinary Portland cement and fly ash are presented in Table 2. The shale ceramsite was crushed shale ceramsite, which was used as coarse aggregate. The properties of shale ceramsite were crushing strength 8.82 MPa, bulk density 842 kg/m^3^ and particle size distribution between 4.75 mm and 19 mm. The sand was nature river sand with properties of modulus of fineness 2.8, density 2650 kg/m^3^ and bulk density 1425 kg/m^3^. The rubber particles were manufactured by mechanical shredding of waste tires. In order to make sure the rubber particles had a similar particle size distribution with sand, the rubber particles used in this research were prepared by artificial formulation. The properties of rubber particles were modulus of fineness 2.7, density 1190 kg/m^3^ and bulk density 365 kg/m^3^. Figure 1 presents the particle size distribution of nature sand, rubber particles and crushed shale ceramsite. The thickener was hydroxypropyl methylcellulose with dosage of 0.04% (in mass of binding material). The water reducer was polycarboxylate superplasticizer with dosage of 1% (in mass of binding material). The water was tap water.

### 2.2. Specimens and Test Methods

According to GB/T 50082-2009 [33], the prism specimens with dimensions 100 mm × 100 mm × 515 mm were prepared for determining the shrinkage values of SCRLC at various rubber particles substitution percentages. Due to good flow of fresh SCRLC, the specimens were manufactured without vibration. After pouring the SCRLC, the specimens were covered with plastic film for 1 day and then demoulded. All specimens were moisture-cured at 20 ± 2 °C and relative humidity > 95% until testing.

**Figure 2:**
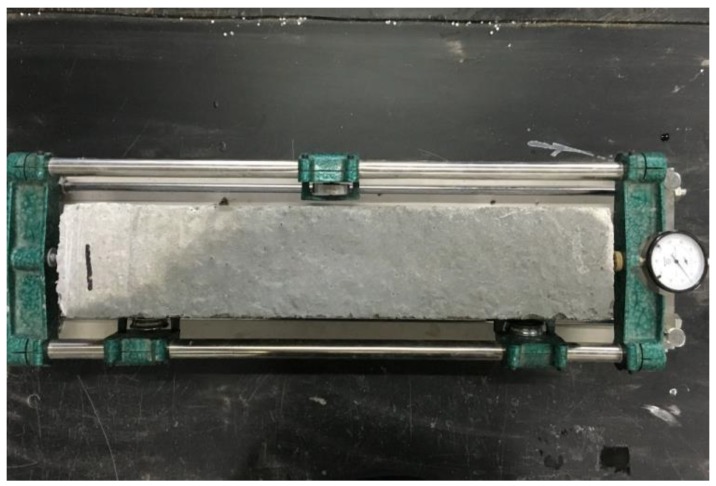
Shrinkage test.

The contact method was utilized to measure the shrinkage value of SCRLC in accordance with GB/T 50082-2009 [33] (as shown in Figure 2). The shrinkage testing apparatus, equipped with a dial gauge at one end, was used to measure the shrinkage strain. Before each test, the gauge was calibrated by the standard bar. After demoulding, the length of specimen was monitored immediately and the value was deemed to initial length of specimen. The testing ages were set as 1 d, 3 d, 7 d, 28 d, 60 d, 90 d, 120 d, 150 d, 180 d and 360 d. During testing, the laboratory environment was 20 ± 2 °C and relative humidity was 60% ± 5%. In order to reduce the test error, the side and direction of specimens were kept the same for each test. The representative shrinkage value for each batch was determined from the mean value of three experimental values at each age. The shrinkage stain was calculated as follows:

(1) $$\varepsilon_{\mathrm{st}}=\frac{L_{0}-L_{t}}{L_{b}}$$

where *ε*_st_ was shrinkage strain at age of *t*, *L*_0_ was initial length of specimen (mm), *L_t_* was the length of specimens at testing time *t* (mm), *L_b_* was gauge length (540 mm).

## 3. Results and Discussion

### 3.1. Experimental Shrinkage Strain

The mean and standard deviation of shrinkage strains of SCRLC at different rubber particles substitution percentage are shown in Table 3. Smaller standard deviation for each batch of SCRLC indicated that three testing values were approximate and the mean value could be used to represent the shrinkage strain of SCRLC. With prolongation of age, the shrinkage strains of SCRLC increased. Most increment of shrinkage strain occurred when the age was less than 120 days, whereafter, the shrinkage strain of SCRLC increased slightly.

**Figure 3:**
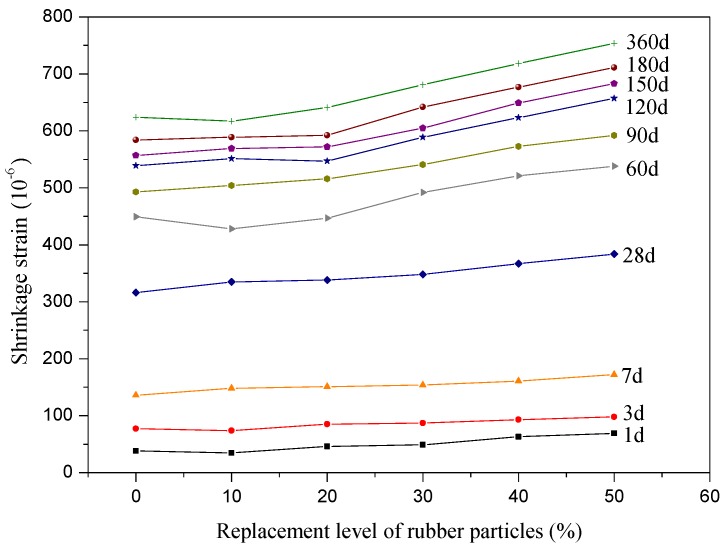
The shrinkage strains of SCRLC versus rubber particles substitution percentages.

Figure 3 illustrates the variation of shrinkage strains of SCRLC at different rubber particles substitution percentages. As the rubber particles substitution percentage increased, the shrinkage strains of SCRLC augmented at each age. The greater the age, the larger the increment of shrinkage strains of SCRLC with an increase of rubber particle substitution percentage would be. When the rubber particles substitution percentage was lower than 10%, the shrinkage strains of SCRLC were similar for each other at different ages. At an age of 360 d, compared with SCLC, the shrinkage strain increased from 624 × 10^−6^ to 754 × 10^−6^ as rubber particles substitution percentage increased from 0% to 50%. This meant that incorporation of rubber particles in SCLC led to a reduction of volume stability of SCLC. This was mainly due to the lower stiffness of rubber particles. When shrinkage deformation occurred during hardening process of cement, rubber particles had lesser resistance deformation ability than sand. It resulted in an increase of shrinkage deformation of SCRLC as rubber particles substitution percentage augmented.

### 3.2. Predictive Models of Shrinkage Strain

#### 3.2.1. Existing Models

Based on the consideration factors of effects of shrinkage deformation of concrete, lots of predictive models have been proposed to evaluate the shrinkage deformation of concrete in previous studies, such as ACI 209 model [25], *fib* Model Code-2010 [26], B3 model [27], GL2000 model [28], Dilger model [29], China Academy of Building Research model [30].

(1) ACI 209 model

ACI 209 model was proposed by American Concrete Institute (ACI) and can be expressed as the following:

(2) $$\varepsilon_{sh}\left(t,t_{sh,0}\right)=\frac{t-t_{sh,0}}{35+(t-t_{sh,0})}\varepsilon_{sh,\infty }\;(\mathrm{moist}\;\mathrm{cured})$$

(3) $$\varepsilon_{sh}\left(t,t_{sh,0}\right)=\frac{t-t_{sh,0}}{55+(t-t_{sh,0})}\varepsilon_{sh,\infty }\;(\mathrm{steam}\;\mathrm{cured})$$

where *ε_sh_(t*, *t_sh,_*_0_*)* is shrinkage strain, *t* is age (d), *t_sh,_*_0_ is curing age at the beginning of drying (d), and *ε_sh,∞_* is final shrinkage strain.

(2) *fib* Model Code-2010

*fib* Model Code-2010, mentioned in International Federation for Structural Concrete, was used to predict the total shrinkage or swelling strains of concrete. In this model, the shrinkage strains could be calculated by the formulas as follows:

(4) $$\varepsilon_{\mathrm{cs}}(t,t_{s})=\varepsilon_{\mathrm{cbs}}(t)+\varepsilon_{\mathrm{cds}}(t,t_{s})$$

(5) $$\varepsilon_{\mathrm{cbs}}(t)=\varepsilon_{\mathrm{cbs}}{}_{0}(f_{cm})\cdot \beta_{bs}(t)$$

(6) $$\varepsilon_{\mathrm{cds}}(t,t_{s})=\varepsilon_{\mathrm{cds}0}(f_{cm})\cdot \beta_{RH}(RH)\cdot \beta_{ds}(t-t_{s})$$

(7) $$\varepsilon_{\mathrm{cbs}}{}_{0}(f_{cm})=-\alpha_{bs}{\left(\frac{0.1f_{cm}}{6+0.1f_{cm}}\right)}^{2.5}\cdot 10^{-6}$$

(8) $$\beta_{bs}(t)=1-\exp(-0.2\sqrt{t})$$

(9) $$\varepsilon_{\mathrm{cds}0}(f_{cm})=[(220+110\cdot \alpha_{ds1})\cdot \exp(-\alpha_{ds2}\cdot f_{cm})]\cdot 10^{-6}$$

(10) $$\beta_{\mathrm{RH}}=\left\{ \begin{array}{l} -1.55(1-{\left(\frac{RH}{100}\right)}^{3})\;for\;40\%\leq RH<99\%\cdot \beta_{s1}\\ 0.25\;for\;RH\geq 99\%\;\cdot \beta_{s1} \end{array}\right.$$

(11) $$\beta_{ds}(t-t_{s})={\left[\frac{\left(t-t_{s}\right)}{0.035\cdot h^{2}+(t-t_{s})}\right]}^{0.5}$$

(12) $$\beta_{s1}={\left(\frac{35}{f_{cm}}\right)}^{0.1}\leq 1.0$$

(13) $$\varepsilon_{\mathrm{lcs}}(t,t_{s})=\eta \cdot \varepsilon_{\mathrm{cs}}(t,t_{s})$$

where *ε_cs_ (t*, *t_s_)* is total shrinkage or swelling strains of NC, *ε_cbs_ (t)* is basic shrinkage of NC, *ε_cds_ (t*, *t_s_)* is drying shrinkage of NC, *t* is the concrete age in days, *t_s_* is the concrete age at the beginning of drying in days, (*t* − *t_s_*) is the duration of drying in days, *ε_cbs0_ (f_cm_)* is the basic notional shrinkage coefficient, *β_bc_* (*t*) is the time function, *f_cm_* is mean compressive strength of concrete at 28 days (MPa), *RH* is relative humidity (%), *α_bs_*, *α_ds_*_1_ and *α_ds2_* are coefficients dependent on the type of cement, respectively, *h* is effective height of section (mm), and *ε_lcs_ (t, t_s_)* is shrinkage of LC, *η* = 1.2 for LC20 and higher.

(3) B3 model

Bazant developed a shrinkage predictive model on account of existing shrinkage and creep data named B3 model. The formulas were shown as

(14) $$\varepsilon_{\mathrm{sh}}(t,t_{s})=-\varepsilon_{\mathrm{sh}\infty }\cdot K_{h}\cdot S(t)$$

(15) $$\varepsilon_{\mathrm{sh}\infty }=\alpha_{1}\alpha_{2}[26{\left(\omega \right)}^{2.1}{\left(f_{c}^{\prime }\right)}^{-0.28}+270]$$

(16) $$K_{h}=1-h^{3}$$

(17) $$S(t)=\tanh\sqrt{\frac{t-t_{0}}{\tau_{sh}}}$$

where *ε*_sh_ (*t*, *t_s_*) is shrinkage strain at time *t* (in/in), *ε*_sh∞_ is ultimate shrinkage strain (in/in), *K_h_* is cross-section shape factor, *S*(*t*) is time function for shrinkage, *α*_1_ and *α*_2_ are parameters related to the type of cement and curing condition, *w* is water content (*lb*/*ft*^3^), *f’_c_* is mean cylinder compressive strength at 28 day, *h* is relative humidity (%), *t* is age of concrete (days), *t*_0_ is age of concrete at beginning of shrinkage, and *τ_sh_* is shrinkage halftime in days.

(4) GL2000 model

Based on the ACI 209 model, GL2000 model was proposed by Gardner and Lockman to describe the shrinkage properties of concrete in 2000. The Equations were given as follows:

(18) $$\varepsilon_{sh}=\varepsilon_{shu}\beta_{RH}\beta_{s}(t,t_{s})$$

(19) εshu=1000⋅βsc⋅(30fc28)12⋅10−6

(20) $$\beta_{RH}=1-1.18RH^{4}$$

(21) $$\beta_{s}(t,t_{s})={\left[\frac{t-t_{s}}{t-t_{s}+0.15{\left(V/S\right)}^{2}}\right]}^{0.5}$$

(5) Dilger model

Dilger model divided the total shrinkage into basic shrinkage and drying shrinkage and calculated the total shrinkage by the following formulae:

(22) $$\varepsilon_{cs}\left(t,t_{s}\right)=\varepsilon_{bs}\left(t\right)+\varepsilon_{ds}\left(t,t_{s}\right)$$

(23) $$\varepsilon_{bs}\left(t\right)=\varepsilon_{bs0}\beta_{bs}\left(t\right)\times 10^{-6}$$

(24) $$\varepsilon_{bs0}=\left\{ \begin{array}{l} 700\times \exp\left(-3.5\times \frac{w}{c}\right)+120\;\mathrm{Concrete}\;\mathrm{with}\;\mathrm{silica}\;\mathrm{fume}\\ 700\times \exp\left(-3.5\times \frac{w}{c}\right)\;\mathrm{Concrete}\;\mathrm{without}\;\mathrm{silica}\;\mathrm{fume} \end{array}\right.$$

(25) $$\beta_{\mathrm{bs}}\left(t\right)=\frac{t^{0.7}}{\gamma_{bs}+\alpha_{bs}t^{0.7}}$$

(26) $$\gamma_{bs}=16.7\times \left(1-\alpha_{bs}\right)$$

(27) $$\alpha_{bs}=1.04-\frac{1}{3}\frac{w}{c}$$

(28) $$\varepsilon_{\mathrm{ds}}\left(t,t_{s}\right)=\varepsilon_{ds0}\beta_{RH}\beta_{\mathrm{ds}}\left(t,t_{s}\right)\times 10^{-6}$$

(29) $$\varepsilon_{ds0}={\left(100\times \frac{w}{c}\right)}^{2}f_{c28}{}^{-0.23}+200$$

(30) $$\beta_{RH}=1.22-1.75\times {\left(\frac{RH}{100}\right)}^{3}$$

(31) $$\beta_{ds}\left(t,t_{s}\right)=\frac{{\left(t-t_{s}\right)}^{0.6}}{0.0016\times {\left(V/S\right)}^{2}\gamma_{\mathrm{ds}}+{\left(t-t_{s}\right)}^{0.6}}$$

(32) $$\gamma_{ds}=6.42+1.5\ln\left(t_{s}\right)$$

(6) China Academy of Building Research model

The China Academy of Building Research model was regressed from numeral experimental data by researchers in concrete research institute, China Academy of Building Research. The predictive formula could be expressed as the following:

(33) $$\varepsilon \left(t\right)=\varepsilon {\left(t\right)}_{0}\cdot \beta_{1}\cdot \beta_{2}\cdot \beta_{3}\cdot \beta_{5}\cdot \beta_{6}$$

where *ε*(*t*)_0_ is the basic equation of shrinkage, *β*_1_ is the influence coefficient of relative humidity, *β*_2_ is the influence coefficient of sectional dimension, *β*_3_ is the influence coefficient of curing method, *β*_4_ is the influence coefficient of fly ash instead of cement, and *β*_5_ is influence coefficient of strength grade of concrete.

The basic equation of shrinkage can be expressed as follows:

(34) $$\varepsilon {\left(t\right)}_{0}=\frac{t}{a+bt}\times 10^{-3}$$

where *a* and *b* are the coefficient regressing from experimental data.

#### 3.2.2. Comparison Analysis

After analyzing the above shrinkage predictive models, it could be detected that the shrinkage prediction value calculated by ACI 209 model, GL 2000 model, *fib* Model Code-2010, B3 model and China Academy of Building Research model were all total shrinkage which included drying shrinkage and autogenous shrinkage. Compared to the above models, it could be seen that China Academy of Building Research model was much simpler than the other models, and the factors considered in this model was much more close to the experimental design. In addition, the China Academy of Building Research model was also utilized to predict the shrinkage strain of LC in JGJ51-2002 [34]. Therefore, the China Academy of Building Research model was tentatively selected to describe the shrinkage properties of SCRLC in this research. According to JGJ51-2002 [34], for LC, *a* = 78.69 and *b* = 1.20 at age of 3 days; meanwhile, *a* = 120.23 and *b* = 2.26 at age of 28 days. It meant that prediction of shrinkage strain of concrete at different ages would utilize different parameters *a* and *b*.

**Figure 4:**
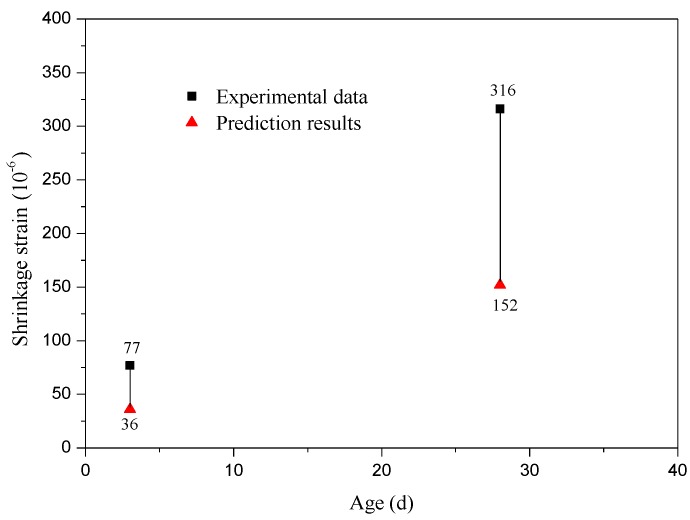
Comparison of shrinkage strain of SCLC between experiment and prediction.

Figure 4 shows the comparison of shrinkage strains of SCLC between experiment and prediction. It can be seen that the experimental results were more than twice as large as predictive results. It indicated that using China Academy of Building Research model to predict the shrinkage strain of SCLC directly was unsuitable. Furthermore, incorporation of rubber particles in SCLC might lead to a much greater difference between SCLC and SCRLC. The gap between the shrinkage strain of SCRLC predicted by China Academy of Building Research model and experimental test values would be much larger. Thus, the China Academy of Building Research model would be also unsuitable to predict the higher shrinkage strains of SCRLC. Proposing a simple predictive model for shrinkage strain of SCRLC was necessary.

#### 3.2.3. Modified Model

Based on the above cases, in order to build a model to describe the experimental results of shrinkage strains of SCRLC, the China Academy of Building Research model was selected as a basic equation to fit the experimental results. On the basis of China Academy of Building Research model, the influence coefficient of rubber particles substitution percentage *β*_6_ was introduced and the predictive formula was exhibited as the following:

(35) $$\varepsilon \left(t\right)=\varepsilon {\left(t\right)}_{0}\cdot \beta_{1}\cdot \beta_{2}\cdot \beta_{3}\cdot \beta_{4}\cdot \beta_{5}\cdot \beta_{6}=\varepsilon {\left(t\right)}_{1}\cdot \beta_{6}$$

**Figure 5:**
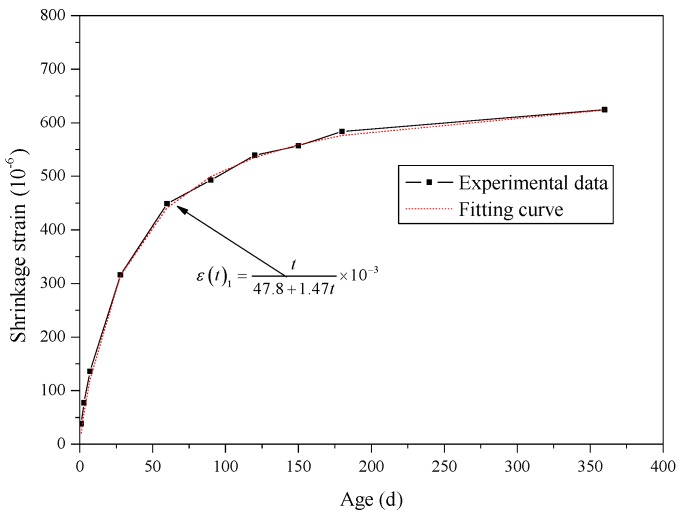
Comparison of fitting curve and experimental curve for basic equation of shrinkage of SCLC.

Through fitting analysis of experimental shrinkage strains of SCLC by MATLAB software (seen in Figure 5), the parameters *a* and *b* in basic equation of shrinkage of SCRLC were 47.8 and 1.47, respectively. The basic equation of shrinkage of SCRLC *ε*(*t*)_1_ could be expressed as follows:

(36) $$\varepsilon {\left(t\right)}_{1}=\frac{t}{0.0478+0.00147t}\times 10^{-3}$$

After comparative analysis, it could be seen that change law of fitting curve was in accordance with the experimental results. The correlation coefficient between the fitting curve and experimental data was 0.9968 which was at a high level. This indicated that Equation (36) would be used as basic equation for shrinkage strain of SCLC.

Considering the effects of rubber particles substitution percentage on the shrinkage strain of SCLC, through statistical regression of experimental data, the influence coefficients of rubber particles substitution percentage *β*_6_ are summarized in Table 4. Then, the predication model for shrinkage strain of SCRLC would be described as the following:

(37) $$\varepsilon \left(t\right)=\frac{t}{47.8+1.47t}\times 10^{-3}\times \beta_{6}$$

**Figure 6:**
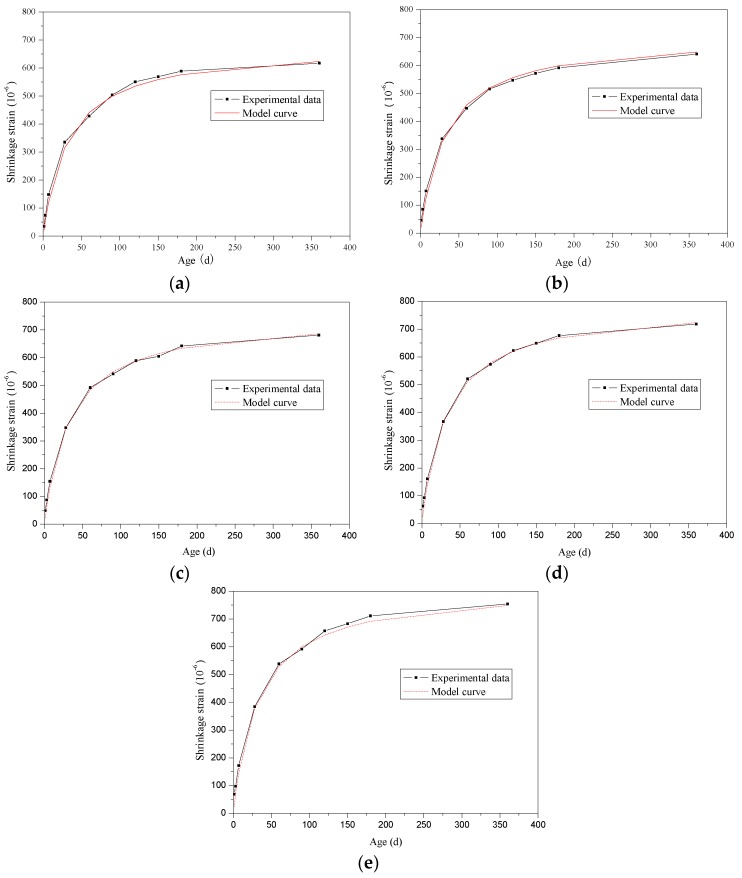
Comparison of shrinkage strains of SCRLC obtained from experiment and fitting: (**a**) SCRLC10; (**b**) SCRLC20; (**c**) SCRLC30; (**d**) SCRLC40; (**e**) SCRLC50.

Figure 6 shows the comparison of shrinkage strains of SCRLC obtained from experiment and fitting. It can be clearly seen that the fitting values were in good accordance with experimental results for each batch. This indicated that as selection of appropriate influence coefficients of rubber particles substitution percentage *β_6_*, the proposed model could be used to describe the shrinkage strains of SCRLC.

**Figure 7:**
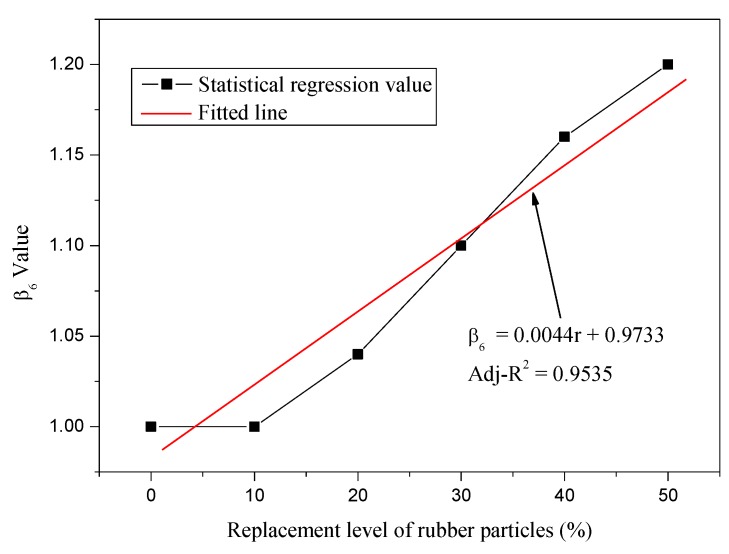
Relationship between *β*_6_ and *r*.

The relationship between *β*_6_ and rubber particles substitution percentage *r* was established as shown in Figure 7. It could be observed that the relationship between *β*_6_ and *r* was approximately linear with correlation coefficient of 0.9535, and the regression equation could be expressed as follows:

(38) $$\beta_{6}=0.0044r+0.9733$$

#### 3.2.4. Shrinkage Strain Prediction

**Figure 8:**
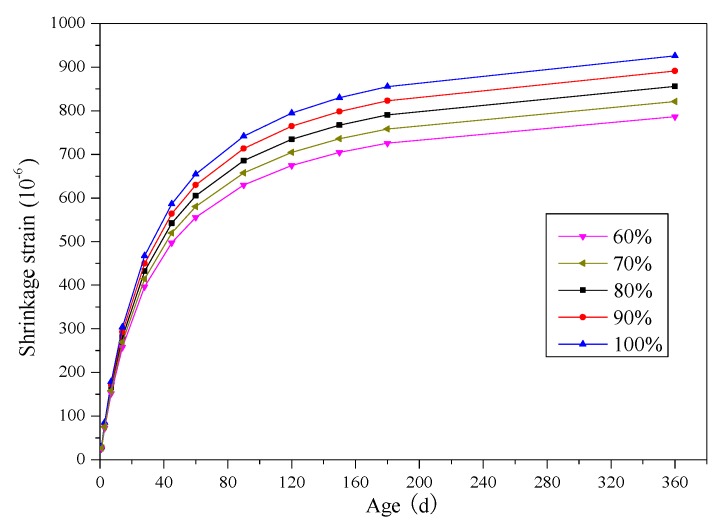
Mean shrinkage strain of SCRLC obtained from predictive model.

Based on the relationship between *β*_6_ and *r* described by Equation (38), the mean shrinkage strains of SCRLC with rubber particles substitution percentage estimates varied from 60% to 100%; these are presented in Figure 8. When the rubber particles substitution percentage increased from 60% to 100%, the shrinkage strains of SCRLC increased for each batch at a different age. According to GB/T 50082-2009 [33], the shrinkage strain of concrete at age of 360 days was deemed as final shrinkage. For SCRLC, with rubber particles substitution percentage increased from 0% to 100%, the final shrinkage strain increased from 624 × 10^−6^ to 894 × 10^−6^ with an increment of 48.4%. Nevertheless, the limiting value of shrinkage strain for LC was 820 × 10^−6^, which was stipulated in JGJ51-2002. When the rubber particles substitution percentage was 80%, the shrinkage strain of SCRLC was 834 × 10^−6^, which exceeded the limiting value. This meant that more than 80% of rubber particles substitution percentage in SCRLC would seriously affect the durability of SCRLC and should be given more attention in application of actual engineering.

### 3.3. Discussion

The shrinkage properties of concrete were closely related to the characteristics of aggregate and the water to cement ratio [35]; the higher the water to cement ratio, the larger shrinkage strain would be, while the higher stiffness of aggregate, the smaller shrinkage strain would be. In this investigation, the rubber particles were utilized to substitute the sand in SCLC. Compared with sand, the stiffness of rubber particles was much lower. Shrinkage occurred during the setting and hardening process of cement and the deformation of rubber particles was much larger than sand. Therefore, keeping material composition of SCRLC the same, except the ratio of rubber particles to sand, the shrinkage of SCRLC increased as rubber particles substitution percentage raised. In order to obtain smaller shrinkage strain of SCRLC and maintain excellent durability of SCRLC, a reduction of the water to cement ratio in SCRLC should be accompanied with an increase of the rubber particles substitution percentage. Moreover, incorporation of expansive agent in SCRLC was also a way to reduce the shrinkage deformation. By comprehensive consideration, the mix proportion of SCRLC should be confirmed by actual demand and the rubber particles substitution percentages should be controlled at reasonable range. Therefore, the SCRLC would be applied in practical engineering and a huge environmental benefits would be achieved in the future.

## 4. Conclusions

Based on the experimental results of shrinkage properties of SCRLC and contrastive analysis of the existing prediction models of shrinkage strains of concrete, the shrinkage properties of SCRLC were studied and the prediction model of shrinkage strain of SCRLC was proposed in this investigation; the conclusions could be drawn as follows:

(1) The shrinkage strains of SCRLC increased with an increase of rubber particles substitution percentages. Most increment of shrinkage strain of SCRLC occurred during the age of 3–120 days. Compared with the plain sample, the shrinkage strain of SCRLC increased from 624 × 10^−6^ to 754 × 10^−6^ as rubber particles substitution percentage increased from 0% to 50% at an age of 360 days.
(2) Based on the China Academy of Building Research model, the influence coefficients of rubber particles substitution percentage *β*_6_ were introduced and a model was established to depict the shrinkage properties of SCRLC. After statistical regression, the influence coefficients of rubber particles substitution percentage *β*_6_ were calculated.
(3) According to the relationship between coefficients of rubber particles substitution percentage and rubber particles substitution percentage, shrinkage strains of SCRLC with rubber particles substitution percentages ranged from 60% to 100% and were predicted by the proposed model. When the rubber particles substitution percentage was larger than 80%, the shrinkage strain of SCRLC at age of 360 days was bigger than 820 × 10^−6^, which exceeded the limiting value stipulated in JGJ51-2002.

## Figures and Tables

**Table 1:** Mix proportions for concrete.

Type of Concrete	Replacement (by Volume)	Weight per Cubic Meter (kg/m^3^)							
		Cement	Fly Ash	Rubber Particles	Sand	Shale Ceramsite	Thickener	Water Reducer	Water
SCLC	0	425	85	0	700	610	0.204	5.1	179
SCRLC10	10%	425	85	31	630	610	0.204	5.1	179
SCRLC20	20%	425	85	62	560	610	0.204	5.1	179
SCRLC30	30%	425	85	93	490	610	0.204	5.1	179
SCRLC40	40%	425	85	124	420	610	0.204	5.1	179
SCRLC50	50%	425	85	155	350	610	0.204	5.1	179

**Table 2:** Chemical compositions and physical properties of ordinary Portland cement and fly ash.

Chemical Analysis (%)	Ordinary Portland Cement	Fly Ash
CaO	62.45	5.31
SiO_2_	20.18	48.92
Al_2_O_3_	4.91	26.27
Fe_2_O_3_	3.88	5.86
MgO	2.67	0.84
SO_3_	2.14	1.21
K_2_O	0.47	0.79
Na_2_O	0.29	0.22
Loss on ignition	2.05	3.60
Specific gravity	3.16	2.21
Fineness (m^2^/kg)	331	275

**Table 3:** Mean and standard deviation of shrinkage strains of SCRLC.

Type of Concrete	Parameter	Ages (d)									
		1	3	7	28	60	90	120	150	180	360
SCLC	Mean value (10^−6^)	38	77	136	316	449	493	539	557	584	624
	Standard deviation	3.6	7.9	4.4	7.2	7.5	12.2	20.0	21.9	13.9	9.2
SCRLC10	Mean value (10^−6^)	35	74	148	335	428	504	551	569	589	617
	Standard deviation	5.7	7.0	16.1	9.6	20.7	21.0	16.7	11.1	11.5	18.5
SCRLC20	Mean value (10^−6^)	46	85	151	338	447	516	547	572	592	641
	Standard deviation	7.0	5.6	12.1	16.5	13.1	18.5	4.6	18.4	16.5	12.1
SCRLC30	Mean value (10^−6^)	49	87	154	348	492	541	589	605	642	681
	Standard deviation	5.6	7.2	7.2	15.9	13.5	9.6	4.6	17.1	8.2	15.7
SCRLC40	Mean value (10^−6^)	63	93	161	367	521	573	623	649	677	718
	Standard deviation	4.4	4.5	11.5	14.5	14.0	7.9	13.2	8.7	14.5	15.1
SCRLC50	Mean value (10^−6^)	69	98	172	384	538	592	657	683	711	754
	Standard deviation	5.6	4.0	7.8	13.1	8.7	8.2	17.7	17.1	23.5	24.1

**Table 4:** Influence coefficient of rubber particles substitution percentage.

Influence Factor	Rubber Particles Substitution Percentage (%)	Symbol	Influence Coefficients
Rubber Particles Substituted Sand	0	*β_6_*	1.00
	10		1.00
	20		1.04
	30		1.10
	40		1.16
	50		1.20
